# Exploring Chemoprevention in Colorectal Cancer for Patients with Inflammatory Bowel Disease: Mechanisms of Action and Clinical Aspects

**DOI:** 10.3390/cancers17020229

**Published:** 2025-01-12

**Authors:** Fotios S. Fousekis, Konstantinos Mpakogiannis, Panagiotis Filis, Alexandros Skamnelos, Dimitrios K. Christodoulou, Davide Mauri, Konstantinos H. Katsanos

**Affiliations:** 1 Department of Gastroenterology, University Hospital of Ioannina, 455 00 Ioannina, Greece; kostismpakogiannis@gmail.com (K.M.); alskamnelos@gmail.com (A.S.); dchristodoulou@gmail.com (D.K.C.); khkostas@hotmail.com (K.H.K.); 2 Department of Medical Oncology, School of Health Sciences, Faculty of Medicine, University of Ioannina, 455 00 Ioannina, Greecedvd.mauri@gmail.com (D.M.)

**Keywords:** inflammatory bowel disease, colorectal cancer, chemoprevention, Crohn’s disease, ulcerative colitis

## Abstract

**Background:** Inflammatory bowel diseases (IBDs) have been associated with a higher risk of colorectal cancer (CRC) development and chronic colonic inflammation seems to have a critical role in the pathogenesis of CRC in patients suffering from IBD. In respect to that, surveillance colonoscopy at regular intervals is recommended in patients with colitis. **Objective:** This review aims to explore the chemopreventive potential of a range of agents, including mesalazine, thiopurines, anti-TNF agents, statins, ursodeoxycholic acid, aspirin, folic acid, and nutraceuticals. **Results:** These agents target inflammation, oxidative stress, and oncogenic pathways, thereby offering the potential to reduce the risk of CRC in patients with IBD. Anti-TNF agents, such as infliximab and adalimumab, not only reduce colonic inflammation, but also play a protective role against CRC by lessening the carcinogenic effects associated with prolonged inflammatory processes. Furthermore, mesalazine and thiopurines have demonstrated established efficacy, while newer biologics, including interleukin inhibitors, show promising advancements. Although nutraceuticals and dietary interventions require further clinical validation, they offer additional possibilities for non-pharmacological prevention. **Conclusion:** Despite progress, knowledge gaps persist regarding the long-term safety, optimal dosing, and combined use of these agents. A significant reduction in the incidence of CRC in patients with IBD could be achieved by advancing chemoprevention and personalizing strategies.

## Simple Summary

Patients with inflammatory bowel diseases (IBD) are at an increased risk of colorectal cancer (CRC) due to the chronic inflammation in the colon. This review examines potential strategies to prevent CRC in these patients, focusing on medications such as mesalazine, thiopurines, anti-TNF agents, and statins, as well as nutraceuticals and dietary interventions. These approaches aim to address inflammation and other mechanisms related to cancer in order to reduce CRC risk. Although many treatments show promise, further studies are needed to refine dosages, assess long-term safety, and investigate the combined use of different therapies. Regular colonoscopy and personalized prevention strategies are essential for lowering cancer risk and improving outcomes.

## 1. Introduction

Recent data have suggested that chronic inflammation not only damages DNA by producing reactive oxygen and nitrogen species but also creates an environment that promotes carcinogenesis [1,2]. Ongoing inflammation may disrupt normal DNA repair processes, resulting in genomic instability and the accumulation of mutations [3]. Furthermore, disturbances in immune surveillance mechanisms—such as the suppression of cytotoxic T cells and the recruitment of immunosuppressive regulatory T cells (Tregs) and myeloid-derived suppressor cells (MDSCs)—may facilitate the invasion and metastasis of cancer cells, thereby further contributing to tumor progression [2]. In this setting, patients suffering from inflammatory bowel diseases (IBDs) have a higher risk of colorectal cancer (CRC) development, particularly patients with ulcerative colitis (UC) compared to the general population. The incidence of CRC in IBD patients varies among different studies and depends on the duration, location, and activity of the disease. Patients with extensive colitis, a long-term duration, an active disease, and the coexistence of primary sclerosing cholangitis (PSC) seem to carry the highest risk of developing CRC [4]. A meta-analysis involving 116 studies with UC patients demonstrated that the estimated prevalence of CRC in UC is 3.7% and the estimated risk of CRC development 10, 20, and 30 years after UC diagnosis is 2%, 8%, and 18%, respectively [5]. However, other studies have demonstrated a lower risk of CRC development in patients with IBD. A Danish population-based cohort study found that the probability of CRC developing in patients with UC was 0.4%, 1.1%, and 3.1%, after 10, 20, and 30 years of disease, respectively. A recent cohort study of 96.447 patients with UC from Sweden and Denmark demonstrated that patients with UC are at increased risk of CRC development (HR: 1.66), are diagnosed with less advanced CRC, and carry an increased risk of dying of CRC (HR: 1.59) compared to the general population [6]. Another Scandinavian population-based cohort study of 45.035 patients with CD reported that patients with CD are also at higher risk of CRC developing (HR: 1.40) compared to the general population [7]. Overall, the variation in CRC risk reported in the literature may reflect differences in study design, population characteristics, and healthcare systems. Nonetheless, timely monitoring and preventive strategies are crucial for reducing the burden of IBD-associated CRC.

In patients with UC, dysplastic lesions and invasive cancers often manifest as multiple, superficially widespread lesions. Furthermore, these neoplastic lesions are often developed in the areas of the colon with the most severe inflammation [8]. The carcinogenesis pathway of CRC in patients suffering from IBD seems to be different compared to sporadic CRC [9]. It has been suggested that four main factors may play a role in the development of IBD-associated CRC. Chronic inflammation induces increasing epithelial turnover in the colonic mucosa, leading to an increased probability of replicative errors. In colonic biopsies of patients with active IBD, high rates of mitosis have been demonstrated [10]. Secondly, chronic inflammation influences the expression of oncogenic genes, such as β-catenin and the Wnt pathway [11]. Thirdly, chronic inflammation in IBD increases oxidative stress, resulting in DNA damage and the activation of signaling pathways that affect cell differentiation proliferation and apoptosis, eventually promoting carcinogenesis [12]. Finally, specific cytokines of IBD, such as tumor necrosis factor (TNF) and interleukin-6 and -23 appear to promote CRC development [13,14,15].

The genetic landscape of colitis-associated cancer shows distinct mutations when compared to sporadic colorectal cancer. These differences are primarily driven by the chronic inflammation associated with IBD [16]. In colitis-associated colorectal cancer, mutations seem to occur earlier. Chronic inflammation in IBD accelerates mutagenesis in the colonic epithelium, contributing to the replacement of normal cells with non-dysplastic but tumorigenic clones, a phenomenon known as “field cancerization” [17,18]. This process increases the mutational burden in non-dysplastic IBD colon tissue compared to healthy colon tissue [19]. SMAD4, TP53, and KRAS mutation are commonly detected in CRC arising from colitis, while APC mutations appear to be significantly lower in sporadic CRC [20,21]. Furthermore, APC mutations demonstrate late in colitis-associated CRC. On the other hand, TP53 mutations appear early in IBD-associated CRC, contrasting with the later occurrence of these mutations in sporadic CRC, suggesting that alternative mechanisms drive tumorigenesis in colitis-associated cancer [16]. In recent years, several studies have highlighted the role of Nuclear Factor Erythroid 2–Related Factor 2 (NRF2), a transcription factor that plays a central role in cellular defense against oxidative stress, both in the development of IBD and in its potential role in CRC development in individuals with IBD [22,23]. More specifically. NRF2 plays a critical role in colitis-associated cancer, exhibiting a context-dependent dual function: it protects against tumor initiation but may promote tumor progression in advanced stages. In the early stages of colitis-associated cancer, NRF2 prevents DNA damage and the accumulation of mutations by combating oxidative stress [23]. Studies in mice have shown that NRF2-deficient mice are more susceptible to colitis and CRC due to increased oxidative damage and inflammation [23,24]. In the advanced stages of colitis-associated cancer, the hyperactivation of NRF2 enhances cancer cell survival, proliferation, and chemotherapy resistance. This is due to NRF2’s role in metabolic reprogramming and tumor antioxidant defenses, allowing cancer cells to thrive [25]. Aberrant NRF2 activation often results from mutations in its regulator, KEAP1, or from oncogenic signaling pathways [26].

**Figure 1:**
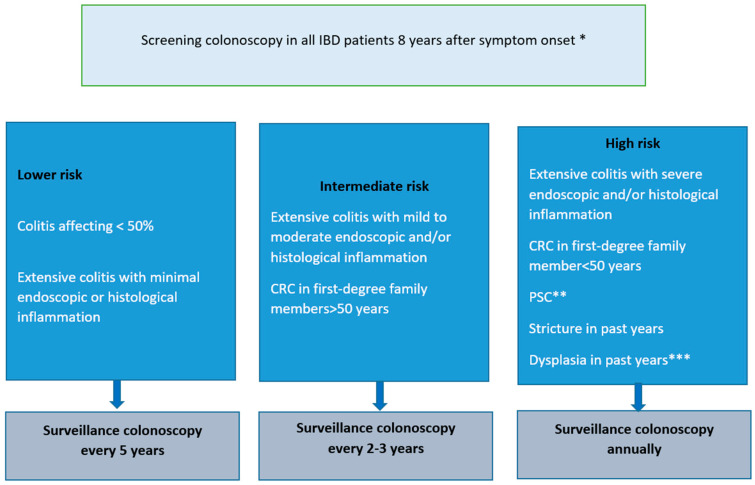
Colonoscopy surveillance in IBD patients according to ECCO guidelines [28]. If possible, surveillance should be performed during disease remission. * In patients who have no colonic involvement or a disease limited to the rectum, no further IBD-specific surveillance is indicated; ** Including post liver transplant; *** In patients who have not undergone surgery.

In respect to that, surveillance colonoscopy is recommended in patients with colitis, while the intervals are determined based on the duration and extension of colitis and the endoscopic findings (Figure 1) [27,28]. On the other hand, chemoprevention is a potential strategy to decrease the prevalence of IBD-related CRC, arresting or reversing the process of colorectal carcinogenesis. Currently, several medications have been developed for the treatment of both CD and UC and may be considered chemopreventive via the inhibition of inflammation.

In this review, we aim to provide a comprehensive analysis of chemopreventive agents in the context of IBD-associated CRC, with a particular focus on their mechanisms of action, to address gaps in the literature. Specifically, we are investigating the interaction between chronic inflammation, chemoprevention, and CRC development, with a focus on the roles of both established and emerging agents, including nutraceuticals and biologics. In this way, we aim to provide novel insights and guidance to chemoprevention strategies in this high-risk population.

## 2. Search Strategy

A comprehensive literature search was conducted using the PubMed and MEDLINE databases to provide an overview of this field, focusing on articles published in English up to December 2024. The keywords and search phrases included “chemoprevention AND colorectal cancer AND inflammatory bowel disease”, “biological agents AND colorectal cancer AND inflammatory bowel disease”, “Ursodeoxycholic acid AND colorectal cancer AND inflammatory bowel disease”, “diet AND colorectal cancer AND inflammatory bowel disease”, and “pathogenesis AND colorectal cancer AND inflammatory bowel disease”. This strategy ensured a broad collection of studies relevant to the chemoprevention of CRC in patients with IBD. Additionally, the search aimed to evaluate the mechanisms of action of agents with potential protective effects against CRC.

The inclusion criteria for the studies were as follows: (i) randomized controlled trials and cross-sectional studies that addressed the research objectives; (ii) original studies and review articles that offered insights into the potential mechanisms of action of chemoprotective agents against colorectal cancer (CRC); and (iii) studies published in English. The exclusion criteria included: (i) studies not published in English and (ii) studies that provided insufficient or unclear data to assess outcomes.

## 3. Potential Chemoprotective Agents

Several agents have been investigated for their potential chemopreventive effects in reducing the risk of colorectal cancer in patients with inflammatory bowel disease. These agents act through different mechanisms, targeting pathways such as inflammation, oxidative stress and oncogenic signaling (Table 1). Below we review these agents in detail.

### 3.1. Mesalazine

Mesalazine, also known as 5-aminosalicylic acid (5-ASA), is widely used in the treatment of IBD, particularly in UC. Its pharmacological profile extends beyond inflammation control and mesalazine appears to have chemopreventive properties in CRC-associated IBD. Mesalazine has several mechanisms of action that contribute to its potential as a chemopreventive agent. It may inhibit β-catenin, preventing its nuclear translocation and the activation of oncogenes involved in cell proliferation. In addition, mesalazine modulates the cyclooxygenase (COX) and lipoxygenase (LOX) pathways, reducing the levels of pro-inflammatory mediators such as prostaglandins and leukotrienes [29]. These pathways are not only associated with inflammation but are also involved in cancer progression. Moreover, mesalazine seems to have antioxidant activity, scavenging reactive oxygen species (ROS) that contribute to DNA damage and tumorigenesis. It also activates the peroxisome proliferator-activated receptor gamma (PPARγ), promoting cell differentiation and further inhibiting cancer cell growth [30]. Lastly, mesalazine has the ability to selectively induce apoptosis in cancer cells without harming normal cells, which underscores its potential as a chemopreventive agent [31,32].

In the most recent and comprehensive meta-analysis, with thirty-one independent observational studies, comprising 2137 cases of colorectal neoplasia (76% of which were cancers), a protective association between the use of 5-aminosalicylates and colorectal neoplasia was found. The analysis demonstrated a reduced risk with a relative risk (RR) of 0.57, indicating a 43% reduction. This significant association was observed in cohort studies (RR = 0.65), case–control studies (RR = 0.53), population-based studies (RR = 0.70), and hospital-based studies (RR = 0.46). In UC, there was a significant risk reduction for colorectal neoplasia (RR = 0.50) and mesalazine use was found to be protective (RR = 0.70) with evidence of a dose effect. However, in Crohn’s disease, the risk reduction was not significant [49]. However, further research is needed to explore the optimal dosing, the duration of mesalazine exposure, and its long-term effects in relation to the severity and progression of the disease.

### 3.2. Thiopurines

Thiopurines are indicated for the maintenance of remission in patients with steroid-dependent UC or intolerance to 5-ASA and as a maintenance therapy in CD [33,34]. While their use is associated with an increased the risk of other malignancies, such as lymphoma, non-Hodgkin’s lymphoma, nonmelanoma skin cancers, and cervical cancer, thiopurines have been shown to have a protective role in the development of colorectal neoplasia in IBD patients. A meta-analysis οf 24 observational studies involving 76,999 participants evaluated the effect of thiopurine use on the risk of colorectal neoplasia in IBD patients. The pooled odds ratio (OR) was 0.63, indicating a 37% reduction in the risk of colorectal neoplasia with thiopurine exposure. The protective effect was particularly significant in patients with UC (OR = 0.67), but not in those with CD (OR = 1.06). Furthermore, thiopurines significantly decreased the risk of colorectal cancer (CRC) (OR = 0.65) and advanced colorectal neoplasia (CRC and/or high-grade dysplasia) (OR = 0.62), although their effect on dysplasia alone was not significant (OR: 0.90) [50]. Another meta-analysis of eleven cohort and 16 case–control studies involving 95397 patients found that the use of thiopurines was associated with a reduced risk of colorectal neoplasia in both case–control studies [OR: 0.49] and cohort studies [RR = 0.96]. Furthermore, the analysis confirmed the chemopreventive effect of thiopurines in patients with a long disease duration (>8 years), but not in those with extensive colitis or primary sclerosing cholangitis, when considering patients at high risk for colorectal neoplasia [51]. However, thiopurine use appears to not have a significant protective effect on the progression of low-grade dysplasia in patients with IBD. In a meta-analysis of five studies comprising 776 IBD patients with low-grade dysplasia, thiopurines (HR = 0.64) did not significantly reduce the risk of advanced colorectal neoplasia (high-grade dysplasia/cancer) in IBD patients with low-grade dysplasia [52].

### 3.3. Anti-TNF Agents

Anti-TNF agents, including infliximab, adalimumab, certolizumab, and golimumab, are biologic therapies widely used for the induction and maintenance of remission in both CD and UC. These agents function by neutralizing tumor necrosis factor-alpha (TNF-α), a key cytokine involved in driving inflammation in IBD; reducing inflammation; and promoting mucosal healing [33,34]. Anti-TNF agents have also shown potential in preventing colitis-associated cancers in animal models by reducing chronic inflammation. In a study using C57BL/6 mice exposed to dextran sulfate sodium (DSS), infliximab administered early in the disease process significantly reduced colorectal tumor formation from 75 to 80% in control mice to 16.7% in treated mice [53]. A recent study using electronic medical records from U.S. hospitals between 1999 and 2020 found that patients with IBD who were treated with anti-TNF agents had a lower likelihood of developing CRC. After adjusting for various factors, the odds ratio (OR) for CRC development in CD patients treated with anti-TNF agents was 0.69 and, for UC patients, the OR was 0.78 [54]. In a Dutch study of IBD patients diagnosed with CRC, anti-TNF therapy was shown to have a significant protective effect against CRC development. Patients treated with anti-TNF agents had a markedly reduced risk of IBD-related CRC, with an odds ratio (OR) of 0.09 [55]. In a case–control study conducted using the Québec health insurance database in Canada, among the 19,582 eligible patients who were treated with anti-TNF agents, there was no evidence of an elevated risk of CRC, suggesting the safety of these biologics regarding cancer development [56].

### 3.4. Statins

Statins are widely used for hypercholesterolemia, inhibiting 3-hydroxy-3-methyl-glutaryl-coenzyme A (HMG-Co A) reductase, which not only reduces cholesterol synthesis but also decreases the production of other important compounds in the mevalonate pathway, such as farnesyl pyrophosphate (FPP) and geranylgeranyl pyrophosphate (GGPP) [35]. These compounds are essential for modifying and activating various cellular proteins, including RAS and RHO, which play a role in carcinogenesis [36,37]. In addition to their HMG-CoA reductase inhibition, statins exhibit various HMG-CoA reductase-independent mechanisms contributing to their pleiotropic effects. These include antioxidant activity, anti-angiogenic [38] and pro-apoptic effects [39], and effects on cell adhesion [40]. Moreover, HMG-Co-A appears to be over-expressed in many types of cancers, including CRC [41]. Experimental data suggest that statins may function as anti-neoplastic agents in colorectal cancer. In this setting, statins have shown growth-inhibitory and pro-apoptotic effects in multiple human colorectal cancer cell lines both in vitro and in tumor xenograft models [57,58]. In this setting, a recent systematic review suggested that statins use may have a role in CRC prevention and treatment [59].

Regarding the chemopreventive effect of statins in patients with IBD, the data are controversial (Table 2). A population-based cohort study demonstrated that the use of statins is not associated with a reduced risk of CRC development (aHR: 0.48, 95% CI: 0.14–1.59, *p*: 0.227) in Chinese patients with IBD [60]. Furthermore, another cohort study from the USA, with IBD patients undergoing colonoscopic surveillance for dysplasia and CRC, found that statin use was not associated with a decreased risk of high-grade dysplasia or CRC development (aHR: 0.63; 95% CI: 0.14–2.90) [61]. On the other hand, a cohort study from the USA found that statins are inversely associated with CRC in IBD patients (OR: 0.42) [62]. In addition, a case–control study found that long-term statin use is associated with lower risk of IBD-related CRC (OR: 0.07) [63]. Another IBD nationwide cohort study from Sweden identified 5273 statin users and 5273 non-statin users, finding that statin use was associated with a lower risk of incident CRC, CRC-related mortality, and all-cause mortality. It is worth noting that the benefit was duration-dependent, with a notably lower risk after two years of statin use [64]. A recent meta-analysis of prognostic factors for advanced colorectal neoplasia in patients with IBD provided weak evidence for the use of statins as chemoprevention (HR: 0.64) [65]. These findings highlight the need for further investigation in order to determine the role of drug type, dose, and duration.

### 3.5. Aspirin

Aspirin, or acetylsalicylic acid, is commonly used as an analgesic, antipyretic, and for cardiovascular prophylaxis. In addition, research has shown that aspirin has the potential to prevent colorectal cancer and other types of cancer [66]. Acetylsalicylic acid seems to have anticancer effects through several mechanisms, including the inhibition of prostaglandin synthesis and WNT–β-catenin signaling, as well as the inactivation of platelets and immune responses. At higher doses, aspirin blocks prostaglandin-endoperoxide synthase 2 and prevents the conversion of arachidonic acid to PGE2, which is implicated in colorectal tumorigenesis [42].

In a recent network meta-analysis of randomized clinical trials with 92550 individuals, there was a statistically significant reduction in colorectal cancer incidence in the high-dose aspirin (500–1200 mg/day) group compared with the group that received no aspirin or a placebo (OR 0.69; 95 per cent credible interval 0.50 to 0.96; surface under the cumulative ranking 0.82). However, this study did not show a statistically significant risk reduction in colorectal cancer incidence with mid- (164–325 mg/day) and low-dose (50–163 mg/day) aspirin [67]. Regarding IBD patients, a meta-analysis with 1282 patients with IBD taking aspirin demonstrated no chemopreventive effect for CRC [pooled OR: 0.66 (95%CI: 0.06–1.39)] [68].

### 3.6. Folic Acid

Vitamin B9, also called folate or folic acid, is a water-soluble vitamin that participates in the synthesis of thymidine and purines, playing an important role in DNA synthesis and replication [69]. Furthermore, it has been suggested that folic acid may improve the chronic inflammation in inflammatory diseases by an increase in some CpG sites of pro-inflammatory genes, leading to the decreased expression of cytokines and chemokines [70]. The relationship between folate status and CRC development seems to be complicated, depending on several factors. In neoplastic cells, the DNA replication occurs at an accelerating rate and the interpretation of the folate pathway may cause ineffective DNA synthesis. This mechanism is the basis of several anticancer agents, such as methotrexate and 5-fluoracil. On the other hand, evidence suggests that folate deficiency in normal epithelial cells may predispose them to neoplastic transformation [71]. Mechanistically, the dose and timing of folate intervention seems to be crucial and supplementation of folic acid after microscopic neoplastic foci may promote the progression of carcinogenesis, while folic acid administration in patients without established neoplasms may be chemopreventive. It is also worth mentioning that a supra-physiological folic acid concentration have been associated with the induction of aberrant DNA methylation in normal human cells in vitro [72].

A meta-analysis evaluated the association between folic acid intake and CRC, demonstrating no benefits in terms of CRC risk in the meta-analysis of both in randomized clinical trials [RR: 1.07 (95% CI: 0.86–1.43)] and in cohort studies [RR = 0.96 (95% CI: 0.76–1.21)] [73]. Nevertheless, another meta-analysis found a protective effect for folic acid supplementation on the development of CRC in patients with IBD [pooled HR = 0.58 (95% CI, 0.37–0.80)]. However, there are several significant limitations to this meta-analysis, such as the retrospective design of all studies, the low number of studies, and an inability to control for possible confounding factors [74].

### 3.7. Ursodeoxycholic Acid (UDCA)

Ursodeoxycholic acid (UDCA), also known as ursodiol, is a natural hydrophilic bile acid (less than 4% of total bile acids), which has been used for the treatment of primary biliary cholangitis, the dissolution of gallstones, and the intrahepatic cholestasis of pregnancy and other hepatobiliary disorders. UDCA may decrease cholestasis, reducing hydrophobic bile acids in the biliary tract, stabilizing the biliary bicarbonate umbrella and limiting the intestinal absorption of cholesterol. Furthermore, UDCA may have antioxidant, anti-inflammatory, and cytoprotective properties [43]. UDCA has been used in patients suffering from PSC, contributing to the improvement of liver biochemical tests; however, UDCA use does not improve transplant-free survival. In addition, a very high dose of UDCA (28–30 mg/kg) has been associated with a worsened course of PSC and is not recommended [75].

In the last decade, evidence from animal models has suggested that UDCA may have a chemoprotective role against CRC in patients with IBD via the activation of Erk1/2, the suppression of c-Myc expression, the inhibition of the epidermal growth factor receptor (EGFr), the TGR5-YAP axis, and the regulation of intracellular ROS generation [44,45,46]. In addition, UDCA may suppress the nuclear factor-kappa B (NF-κB) signaling pathway, which regulates the immune response and inflammation and has been implicated in the process of colorectal carcinogenesis [47,48]. However, clinical studies have provided conflicting results (Table 3). Retrospective studies have demonstrated that UDCA use may prevent the development of advanced colorectal adenoma [76,77]. On the other hand, many studies have shown that UDCA does not affect the frequency of CRC development in IBD patients [78,79,80,81]. It is worth mentioning that a high-dose of UDCA has been implicated in a higher risk of CRC. In a randomized placebo-controlled trial, the administration of high-dose UDCA (28–30 mg/kg/day) was associated with a 4.4 times higher risk of colorectal neoplasia in patients with PSC and UC [82]. Two meta-analyses concluded that only a low dosage of UDCA may benefit patients with IBD and PSC, in terms of CRC development [83,84]. Consequently, the appropriate dose is a matter for discussion, while a high dose of UDCA may contribute to carcinogenesis, influencing gut microbiota.

### 3.8. Nutraceutical-Based Chemopreventive Strategies

The role of nutraceuticals as chemopreventive agents in CRC has garnered significant attention in recent years. In exploring strategies for CRC chemoprevention in IBD, it is important to consider dietary compounds and bioactive substances. Several nutraceuticals show anti-inflammatory, antioxidant, and anticancer properties, suggesting they could enhance existing preventive approaches [85,86].

- Cereals and Whole Grains

Cereal-based nutraceuticals, such as whole grains, are abundant in dietary fiber, phenolic compounds, and antioxidants. These bioactive components have been associated with a reduced risk of colorectal cancer potentially by enhancing the gut barrier function, modulating gut microbiota, and improving the gut’s immunological function [87,88,89]. A recent meta-analysis of randomized control trials demonstrated that cereal fiber supplementation is helpful in increasing the short-chain fatty acid (SCFA) concentration [90]. SCFAs appear to have immunoregulatory and exhibit antineoplastic properties, by enhancing apoptosis and decreasing the proliferation of CRC cells [91].

ii. Grape Seed Extracts

Grape seeds are a rich source of polyphenols, particularly proanthocyanidins, which seem to have antioxidant and anti-inflammatory activities [92]. Grape seed extract has been found to inhibit tumorigenesis in CRC models by scavenging reactive oxygen species and downregulating inflammatory mediators [93]. Additionally, grape seed proanthocyanidins have been to shown to inhibit colon cancer-induced angiogenesis by suppressing the expression of vascular endothelial growth factor and angiopoietin 1 [94].

iii. Butyric Acid

Butyric acid, a short-chain fatty acid produced by the fermentation of dietary fibers by gut microbiota in the colon, plays a crucial role in maintaining colon homeostasis. It has been shown to suppress CRC progression by inhibiting cancerous cells through its role as a histone deacetylase inhibitor [95]. Advanced metabolomic and proteomic research has revealed butyrate suppresses the proliferation of CRC cells by targeting pyruvate kinase M2 and metabolic reprogramming [96].

iv. Curcumin

Curcumin, a bioactive compound derived from the dried roots of the turmeric plant Curcuma longa, has gained significant interest for its anti-inflammatory and anticancer properties [97]. In vitro studies conducted on human colon cancer cell lines have demonstrated that curcumin inhibits cellular growth by inducing cell cycle arrest at the G2/M and G1 phases as well as by inducing apoptosis by interacting with multiple molecular targets, primarily via the extrinsic pathway involving TRAIL/Fas signaling and caspase activation [98,99]. Moreover, its ability to enhance the efficacy of standard chemotherapeutics makes curcumin an attractive adjuvant in CRC treatment strategies [100].

v. Dietary Fiber

The protective role of dietary fiber in CRC prevention is well-documented. Fiber may impact carcinogenesis by affecting bile acid metabolism and providing antioxidants from vegetable sources. It can be digested by gut bacteria to produce beneficial compounds like butyrate [101]. A meta-analysis investigated the relationship between dietary fiber intake and specific types of CRC. The results showed that individuals in the highest quartile of dietary fiber intake had a 14% lower risk of proximal colon cancer and a 21% lower risk of distal colon cancer compared to those with the lowest intake [102].

While preclinical studies and animal models provide promising insights into the chemopreventive potential of nutraceuticals in CRC, there is a lack of strong clinical data focused on patients with IBD. To effectively translate these findings into strategies for preventing IBD-associated CRC, further research is needed.

## 4. Study Limitations

This review has several important limitations that warrant consideration. First, the included studies varied significantly in design, sample size, and population characteristics, which may affect the generalizability of conclusions. Differences in disease severity, duration, and treatment regimens could also influence outcomes related to chemopreventive agents. Second, relying on retrospective studies and meta-analyses introduces potential biases, such as recall and selection bias, which compromise the strength of the evidence. Additionally, many mechanisms of action for various agents are derived from preclinical or animal models, raising concerns about their clinical relevance. The rapidly changing landscape of IBD treatments presents another challenge, as newer therapies with potential chemopreventive effects are often underexplored due to limited data. While nutraceutical-based approaches are promising, they currently lack robust clinical evidence specific to IBD-related colorectal cancer. Lastly, methodological variations across studies complicate direct comparisons and the synthesis of results.

## 5. Future Perspectives

The development of new agents targeting interleukins for the treatment of both UC and CD has revolutionized the management of IBD, offering new therapeutic options. Ustekinumab, an interleukin-12 and interleukin-23 inhibitor has been approved for the treatment of UC and IBD, while interleukin-23 inhibitors, risakinzumab, mirikizumab, and guselkumab are in phase 2b or phase 3 clinical studies [103]. There are limited data about the chemopreventive role of anti-interleukin agents; however, agents targeting interleukins may contribute to the prevention of CRC in IBD patients due to the role of interleukins in CRC pathogenesis [104]. Interleukin-23 has been associated with the enhancement of CRC proliferation and invasion [105]. On the other hand, interleukin-12 appears to have an antitumor activity in preclinical models and some clinical data [106,107]. Recent data have suggested that ustekinumab is not associated with a higher risk of new or recurrent cancer in IBD patients with prior malignancy [108]. However, the potential chemopreventive role of ustekinumab is still unclear and further studies are required regarding the role anti-interleukin agents in the chemoprevention of CRC in IBD.

## 6. Conclusions

Considering the increased CRC risk associated with long-term inflammation, CRC chemoprevention in patients with IBD remains a critical aspect of disease management. Various chemopreventive agents, including mesalazine, anti-TNF agents, and statins have shown promise in reducing the risk of CRC in these patients; however, the evidence is still evolving and further research is required to determine optimal dosing regimens, the long-term effects of chemopreventive agents, and the role of biologics. In addition, nutraceuticals and dietary modifications also show promise, although further clinical validation is required. Newer biologic therapies, including interleukin inhibitors, may represent a promising area of research in the field of chemoprevention, although further investigation is required to fully ascertain their potential. In respect to that, regular surveillance colonoscopy remains essential and a cornerstone of CRC prevention, particularly for patients with extensive colitis and/or a long disease duration. As the understanding of the pathogenesis of IBD-associated CRC grows, a more personalized approach to chemoprevention may optimize outcomes for IBD patients. By leveraging advances in molecular biology and genetic profiling, we can develop precision interventions that account for individual risk factors, including genetic predisposition and disease severity.

## Figures and Tables

**Table 1:** The potential chemopreventive agents and their mechanisms of action.

Chemopreventive Agents	Mechanism of Action	References
Mesalazine (5-ASA)	-Inhibition of β-catenin, preventing oncogene activation -Modulation of COX and LOX pathways to reduce pro-inflammatory mediators -Exhibits antioxidant properties by scavenging reactive oxygen species (ROS) -Promotion of apoptosis in cancer cells without harming normal cells	[29,30,31,32]
Thiopurines	-Maintaining inflammation remission	[33,34]
Anti-TNF agents	-Neutralization of TNF-α, reducing inflammation and promoting mucosal healing	[33,34]
Statins	-Inhibits HMG-CoA reductase -Antioxidant activity, anti-angiogenic and pro-apoptic effects -Cell adhesion modulation -Decrease in oncogenic compounds	[35,36,37,38,39,40,41]
Aspirin	-Inhibition of prostaglandin synthesis and WNT–β-catenin signaling -Inactivation of platelets and immune responses -Blocks prostaglandin-endoperoxide synthase 2 and prevents the conversion of arachidonic acid to PGE2	[42]
Ursodeoxycholic acid	-Antioxidant, anti-inflammatory, and cytoprotective properties -Activation of Erk1/2, suppression of c-Myc expression, inhibition of epidermal growth factor receptor, TGR5-YAP axis, and regulation of intracellular ROS generation -Suppression of NF-κB signaling	[43,44,45,46,47,48]

**Table 2:** Key studies regarding chemopreventive role of statins in IBD-associated CRC.

Study Design	Origin of Study	Number of Patients	Outcomes	Study (Year) [Ref.]
Retrospective	Israel	60 IBD Pts with CRC 1861 non-IBD Pts with CRC	Lower risk of IBD-associated CRC OR: 0.07; 95% CI: 0.01–0.78	Sammader et al. (2011) [63]
Retrospective	USA	11,001 IBD Pts	Lower risk of CRC development OR: 0.42; 95% CI: 0.28–0.62	Anathakrishan et al. (2016) [62]
Retrospective	USA	642 IBD Pts	Invariable risk of HGD and CRC aHR: 0.63; 95% CI: 0.14–2.90	Shah et al. (2019) [61]
Retrospective	China	2103 IBD Pts	Invariable risk of CRC aHR: 0.48, 95% CI:0.14–2.59	Mak et al. (2020) [60]
Retrospective	Sweden	5273 IBD pts, statin users; 5273 IBD pts, non-statin users	Lower risk of CRC development aHR = 0.76 (95% CIs: 0.61 to 0.96)	Sun et al. (2023) [64]

aHR: adjusted hazard ratio, CRC: colorectal cancer, IBD: inflammatory bowel disease, HGD: high-grade dysplasia, Pts: patients, OR: odds ratio.

**Table 3:** Key studies regarding chemopreventive role of UDCA in IBD-associated CRC.

Study Design	Origin of Study	Number of Patients	UDCA Dosage	Outcomes	Study [Ref]
RCT	USA	25 UC-PSC Pts receiving UDCA 31 UC-PSC Pts receiving placebo	28–38 mg/kg/day	Higher risk of CRC (HR:4.44; 95% CI:1.30–20.1)	Eaton et al. [82]
RCT	USA	29 UC-PSC Pts receiving UDCA 23 UC-PSC Pts receiving placebo	13–15 mg/kg/day	Lower risk of CRC (RR:0.26; 95% CI: 0.07–0.99)	Pardi et al. [77]
Retrospective-cohort	USA	59 UC-PSC Pts receiving UDCA 18 UC-PSC Pts receiving placebo	9–10 mg/kg/day	Lower risk of colonic dysplasia (aOR:0.14; 95% CI: 0.03–0.64)	Tung et al. [76]
RCT	Sweden	48 IBD-PSC Pts receiving UDCA 50 IBD-PSC Pts receiving placebo	17–23 mg/kg/day	Invariable risk of CRC 13% vs. 16%	Lindstrom et al. [81]
Retrospective-cohort	USA	28 UC-PSC Pts receiving UDCA 92 UC-PSC Pts no UDCA treatment	Mean dose UDCA 9.2 mg/kg/day	Invariable risk of CRC or dysplasia aHR: 0.59; 95% CI:0.26–1.36	Wolf et al. [79]
Retrospective	United Kingdom	130 IBD-PSC Pts receiving UDCA 36 IBD-PSC Pts no UDCA treatment	15–20 mg/kg/day	Invariable risk of CRC RR: 2.22; 95% CI:0.29–17.14	Braden et al. [80]

aHR: adjusted hazard ratio, aOR: adjusted odds ratio, CRC: colorectal cancer, IBD: inflammatory bowel disease, Pts: patients, RCT: randomized control trial, RR: relative risk, UC: ulcerative colitis.
